# Factors Associated with Having Family/Whānau or Close Friends Who Used Alcohol or Other Drugs in Harmful Ways among University Students in New Zealand

**DOI:** 10.3390/ijerph19010243

**Published:** 2021-12-26

**Authors:** Ben Wamamili, Pauline Stewart, Mark Wallace-Bell

**Affiliations:** 1 School of Health Sciences, University of Canterbury, Christchurch 8041, New Zealand; mark.wallace-bell@canterbury.ac.nz; 2 Family Drug Support Aotearoa, Christchurch 8011, New Zealand; paulinestewartphd@gmail.com

**Keywords:** alcohol use, AoD use, other drug use, family/whanau, close friend

## Abstract

The consequences of alcohol and other drug (AoD) use are well documented. This study investigated factors associated with having family/whānau or close friend who used AoD in harmful ways in New Zealand. Data came from a July–August 2020 cross-sectional survey of students from eight universities (*n* = 946). Participants were asked if they had family/whānau or close friends in New Zealand who consumed alcohol or used other drugs (cannabis, ecstasy/MDMA, methamphetamine, cocaine, heroin, prescription drugs, inhalants, or other) in a way that negatively impacted them, their family, or close friends in the last 12 months. Logistic regression assessed associations of having family/whānau or close friend who used AoD harmfully with student characteristics. Of respondents, 36.2% (33.1–39.4) had family/whānau or close friend who had consumed alcohol harmfully, and 42.9% (39.5–46.3) had family/whānau or close friend who had used at least one drug harmfully. Respondents’ age and ethnicity were significantly associated with having family/whānau or close friend who used AoD harmfully. The results suggest widespread harmful AoD use and potentially significant second-hand effects of AoD use in New Zealand. These data can be used to supplement information from traditional in-person surveys of individuals using alcohol and other drug (e.g., the New Zealand Health Survey).

## 1. Introduction

The negative consequences of alcohol and other drug use (legal and illicit) (AoD) on the health, economic, and social aspects of individuals and communities are well documented [1,2]. Alcohol is one of the most widely used substances globally and is part of many cultural, religious, and social practices [3]. Data from the New Zealand Health Survey (NZHS) show that in 2020/21, four in five (78.5%) adults aged 15+ years consumed an alcoholic drink and one in five (19.9%) engaged in hazardous drinking (defined as an established pattern of drinking that carries a high risk of damage to physical or mental health) [4]. The prevalence of hazardous drinking was highest in adults aged 18–24 (32.9%). Men were 2.0 times more likely than women, and Māori (the Indigenous population of New Zealand) were 1.7 times more likely than non-Māori to be hazardous drinkers. The NZHS provides limited data on illicit drug use. However, it reports that 15.3% and 1.0% of adults used cannabis and amphetamine, respectively, in the past year and men were more likely than women, and Māori more likely than non-Māori to have used these drugs [4]. A 2017 report on the global statistics on alcohol, tobacco, and illicit drug use suggests that New Zealand has some of the highest age-standardised rates of amphetamine, cannabis, opioid, and cocaine use dependence in Australasia [5].

The harmful effects of AoD [6,7,8,9] are far reaching, impacting on the user, their loved ones, and communities [10]. A 2011 study of New Zealanders aged 12–80 years (*n* = 3068) found that 24.8% had at least one self-defined heavy drinker in their life, 84% of whom experienced at least one harm (physical, social, economic or psychological) related to the person’s drinking [11]. A second study found people who had moderate-to-high exposure to heavy drinkers (particularly close household contacts) were 2.3–2.8 times more likely than people with no heavy drinkers in their lives to experience lower health status and personal wellbeing, such as decreased ‘usual’ activity; significant pain and discomfort, and anxiety and depression [12]. Another study of New Zealand adults aged 18–70 years (*n* = 1925) found that 15% of men and 12% of women reported experiencing an aggressive act by a partner, and 11% of men and 16% of women reported being aggressive towards a partner in the past two years. Alcohol was involved in more than 25% of incidents [13].

In New Zealand, the Government’s approach to AoD use is set out in the National Drug Policy, which guides policy and practices aimed at preventing and reducing AoD-related harm in the community [10,14]. The current policy emphasises the need for a person-centred intervention and responses to AoD use that reflect the individual drug’s risk profile [10]. The goals of the policy range from total eradication of use and supply (e.g., methamphetamine) to harm minimisation (e.g., alcohol).

The available literature on AoD use is focused on risk factors from an individual user’s level and peers are among the most important risk factors in the development of AoD use in adolescents and young adults [15]. Peers model AoD use behaviours and increase the positive expectancies of AoD use. Experimentation with AoD during adolescence (high school students) and early adulthood (college and university students) often occurs in a social setting [16]. Commencement of university coincides with a period (16–25 years) at which substance use disorders are at their peak in young people [17]. For most students, AoD use provides a forum for meeting new friends, socialising, and peer relationships [18]. 

Previous studies have described the prevalence and impact of AoD use on university (and college) students in New Zealand (hazardous drinking ranged from 68% to 70%) [19,20] and elsewhere [21,22,23]. Poor academic performance; unintentional injuries; physical assault; sexual assault; unsafe sex; memory loss, and property damage are common in students who use AoD [24,25]. These effects are not limited to students who use AoD. Students who abstain from AoD use may experience second-hand effects, including disrupted study time or sleep due to babysitting intoxicated peers or emotional distress from insults or humiliation because of a peer’s AoD use. [26] Data on the prevalence and impacts of AoD use among university students’ family/whānau members or close friends are lacking. The current exploratory study examined the associations between student characteristics (age, gender, and ethnicity) with having family members or close friends who used AoD in a harmful way in the last 12 months. This approach provides novel data by shifting the focus from the individual user to their wider social sphere.

## 2. Materials and Methods

### 2.1. Design and Data Source

Data were obtained from a cross-section of students across New Zealand between 21 July and 18 August 2020. All students enrolled at any of the eight universities (University of Auckland, Auckland University of Technology, University of Canterbury, Lincoln University, Massey University, University of Otago, University of Waikato, or Victoria University of Wellington) were eligible to participate. A convenience sample was used because complete enrolment lists of students were not available from the universities to allow for random sampling. The survey was, however, widely advertised on social media platforms popular with students, including student associations’ Facebook pages, and could be completed anonymously online or on paper. 

Paper questionnaires were distributed by research assistants who were recruited from students at respective universities through Student Job Search [27]. The research assistants were trained to approach students on campus (at cafes, outside libraries and lecture halls) and invite them to complete the survey on paper or online by scanning a QR code using a smartphone. Research assistants were not allowed to complete the questionnaire on behalf of participants, but rather instructed to distribute questionnaires and pens and collect the completed questionnaires when participants indicated they were finished. Participants were required to be aged 18 years or older and able to consent before participating. A total of 946 students participated (171 online, 775 on paper). 

All procedures were performed in compliance with the University of Canterbury Human Ethics Committee (HEC 2020/35) which reviewed and approved this research.

### 2.2. Measures

#### 2.2.1. Demographic Characteristics

Participants were asked their age, which was categorised as 18–24 years, 25–34 years and 35 years or older. Gender was categorised as male, female, and other (includes ‘other’; ‘prefer not to say’ and missing data), and ethnicity was categorised as New Zealand European, Māori, Pasifika, Asian, and Other. Pasifika includes Samoan, Cook Island Māori, Tongan and Niuean, while Asian includes Indian and Chinese.

#### 2.2.2. Alcohol Use

Participants were asked, “Do you have a family member or close friend who you feel drinks alcohol excessively or has in the last 12 months? By this, we mean that their drinking has negatively impacted them, their family or their close friends.” The responses included “Yes”, “No”, “Don’t know” (no responses were obtained for this option), and “Would rather not say”. 

#### 2.2.3. Drug Use

Participants were asked, “Do you have a family member or close friend who in the last 12 months has used any of the following drugs in a way which has negatively impacted them, their family or their close friends?” The responses included “Cannabis”, “Synthetic cannabis”, “Ecstasy or MDMA”, “Methamphetamine (Meth, P)”, “Cocaine”, “Heroin”, “Prescription drugs”, “Inhalants (e.g., sniffing glue, lighter fluid, solvents)”, “Other”, “Don’t know”, “No, I do not have a family member or close friend who has used any of the above”, and “Would rather not say”. 

### 2.3. Statistical Analysis

Descriptive statistics were conducted to assess the overall rates of exposure to harmful AoD use among family/whānau or close friend. Binary logistic regression analysis examined the relationship between age, gender, and ethnicity and having family/whānau or close friends who consumed alcohol harmfully, or used at least one of three most reported drugs (i.e., cannabis, ecstasy or MDMA, and prescription drugs) harmfully. 

Age was coded as (1 = 35 or older; 2 = 25–34, and 3 = 18–24) and gender as (1 = other, 2 = female, and 3 = male). Ethnicity (1 = NZ European, Māori, Pasifika, Asian or Other, and 2 = non-NZ European, non-Māori, non-Pasifika, non-Asian or non-Other). Although participants could select more than one ethnicity, a binary variable was necessary for the purpose of analysis. For this reason, each ethnic group was compared with all other four groups combined (i.e., NZ European vs. non-NZ European). Harmful consumption of alcohol was coded as (1 = yes, 2 = no), and harmful use of drugs (cannabis, ecstasy or MDMA, or prescription drugs) as (1 = yes, 2 = no). All statistical analyses were performed using IBM SPSS Statistics V.27 and two-sided *p* < 0.05 was considered statistically significant. Adjusted odds ratios (AOR) with the associated confidence intervals (95% CI) were reported.

## 3. Results

### 3.1. Participants

The analysis included 946 students. The demographic characteristics of participants are displayed in Table 1.

### 3.2. Alcohol Use

A total of 873 complete responses (92.3% of the sample) (159 online and 714 on paper) were reported. Overall, 36.2% (95% CI: 33.1 to 39.4) had family/whānau members or close friends who had consumed alcohol in a harmful way in the last 12 months, 62.0% did not, and 1.8% would rather not say. There were no significant differences in responses based on survey type (*p* = 0.127) (Table 2).

### 3.3. The Association between Having Family/Whānau or Close Friend Who Consumed Alcohol in a Harmful Way, with Age, Gender, and Ethnicity

A set of binary logistic models predicted the likelihood of having family/whānau or close friend who consumed alcohol in a harmful way (outcome) with age group, gender, and ethnicity (predictors). The overall model was significant, χ^2^ (df = 9, *n* = 857) = 55.097, *p* < 0.001. Only ethnicity made a significant contribution to the model (Table 3). Māori had significantly higher odds of having family/whānau member or close friend who consumed alcohol in a harmful way compared with non-Māori (AOR = 2.50, 95% CI: 1.50 to 4.18).

### 3.4. Other Drug Use

A total of 798 complete responses (84.4% of the sample) were reported. Overall, 42.9% (95% CI: 39.5 to 46.3) reported a family/whānau member or close friend who used at least one drug in a harmful way in the last 12 months. Cannabis (32.7%), ecstasy or MDMA (25.2%), and prescription drugs (10.5%) were the three most reported drugs (Table 4).

### 3.5. The Association between Having Family/Whānau or Close Friend Who Used Drugs (Cannabis, Ecstasy or mdma, or Prescription Drugs) in a Harmful Way with Age, Gender, and Ethnicity

#### 3.5.1. Cannabis

The overall model was significant, χ^2^ (df = 9, *n* = 798) = 96.049, *p* < 0.001; age and ethnicity made a significant contribution to the model (Table 5). Respondents who were aged 25–34 had higher odds than respondents aged 18–24 of having family/whānau or close friend who used cannabis in a harmful way (AOR = 2.08, 95% CI: 1.23 to 3.52). NZ European (AOR = 4.26, 95% CI: 2.48 to 7.32), and Māori (AOR = 4.53, 95% CI: 2.55 to 8.06) had similar odds compared with non-NZ European and non-Māori, respectively. Likewise, Pasifika (AOR = 2.06, 95% CI: 1.08 to 3.92) and Other (AOR = 2.27, 95% CI: 1.29 to 4.00) had similar odds compared with non-Pasifika and non-Other, respectively, of having family/whānau member or close friend who used cannabis in a harmful way.

#### 3.5.2. Ecstasy or MDMA

The model was significant, χ^2^ (df = 9, *n* = 798) = 98.925, *p* < 0.001. Age and ethnicity made a significant contribution to the model (Table 5). Respondents who were aged 25–34 had higher odds than respondents aged 18–24 of having family/whānau or close friend who used ecstasy/MDMA in a harmful way (AOR = 2.88, 95% CI: 1.53 to 5.43). NZ European (AOR = 5.50, 95% CI: 3.04 to 9.96), Māori (AOR = 1.88, 95% CI: 1.06 to 3.35), and Other (AOR = 2.72, 95% CI: 1.47 to 5.03) had higher odds of having family/whānau member or close friend who used ecstasy/MDMA in a harmful way than non-NZ European, non-Māori, and non-Other, respectively. 

#### 3.5.3. Prescription Drugs

The overall model was significant, χ^2^ (df = 9, *n* = 798) = 18.800, *p* = 0.027, but age, gender, and ethnicity did not make a significant contribution to the model (Table 5).

## 4. Discussion

The current study adds to the growing literature on AoD use and its potential effects on tertiary students. Further, the study explores new approaches that move the discourse on AoD use beyond an individual user to their close contacts (family and friends). Consistent with previous population surveys [4], this study found that age and ethnicity were significantly associated with having family/whānau member or close friend who had used AoD harmfully. Overall, 36.2% of respondents had family/whānau member or close friend who had consumed alcohol in a harmful way, and 42.9% had family/whānau member or close friend who had used at least one drug in a harmful way in the last 12 months.

Although the survey questions and methods (online and paper questionnaires) used in this study were exploratory, they appear sound and the findings are comparable to findings of studies that used ‘traditional’ approaches (i.e., in-person interviews with people who use AoD or with people in the general population). For example, Māori participants were more likely than non-Māori to report family/whānau member or close friends who consumed alcohol harmfully, and age and ethnicity were significantly associated with participants reporting cannabis and ecstasy or MDMA use among family/whānau members or close friends, consistent with the NZHS [4]. 

There are considerable differences, however, between the results of the current study and those from the general population. Our results of harmful alcohol consumption (36.2%) and cannabis use (32.7%) were substantially higher compared with the 2020/21 estimates from the NZHS, where 19.9% of adults engaged in hazardous drinking and 15.3% used cannabis in the past year [4]. The differences between these estimates may be due to a number of reasons. Firstly, the NZHS gathers information directly from individual users of AoD whereas the current study used secondary sources (family/whānau members and close friends of users). Under-reporting of alcohol consumption is common in self-reported population surveys [28,29]. Individual users may under-report use due to fear of stigmatisation, and family members may under-disclose AoD use by their loved ones in one-on-one interviews due to concealed stigma [30], which is associated with chronic sorrow among family members and close friends of people using AoD. Secondly, it is possible that some participants in the current study may have nominated the same people: flatmates, classmates, or workmates may have common close friends and may engage in similar activities, including harmful AoD use. 

Another significant finding was that students aged 25–34 years were more likely than those aged 18–24 years to report harmful cannabis use among their family members or close friends, whereas in the general population the prevalence of cannabis use was higher in people aged 18–24 years than 25–34 years (38.2% vs. 23.4%). These differences should be investigated further to understand what they mean and to determine the validity of the survey tools ‘explored’ in the current study. As the AoD use environment continues to evolve, particularly in the face of the COVID-19 pandemic, public health tools must also evolve to address new challenges. Accurate information about AoD use is important for targeted preventative interventions. Data from secondary sources (family, close friends) can help to supplement self-reported data and identify inconsistencies in prevalence estimates from traditional national population surveys. 

While we know that there is a high prevalence of harmful AoD use in New Zealand, it appears that many more people are close to someone with harmful AoD use, and they may be exposed to the harmful effects of that person’s AoD use: about 36% reported exposure to harmful alcohol use and 43% to another drug use in the current study. Previous research reports a wide range of harms, including academic, social, emotional, physical, and financial [26,31,32]. In an educational setting, an intoxicated family/whānau member or close friend may contribute to a student’s poor grades and satisfaction with university or college experience through interrupted study time and sleep (to look after an intoxicated family member, friend, or flatmate) [26]. In addition, students being impacted by AoD use of family and friends could impact student’s financial or other support and/or ongoing family relationships. It could also increase anxiety in students who may worry about particular family members or close friends at times such that focus is diverted from studies. All these harms are nevertheless potentially preventable.

Given the possible negative impact of having a family/whānau member with alcohol and other drug misuse, consideration could be given to how students, especially Māori, could be supported to build coping and resilience while engaged in studies. Telephone support could assist students faced with situations they wish to address, while maintaining anonymity. Psychosocial counselling and support groups could also provide support to students needing help to manage situations which prove challenging, especially if such support was available through Telehealth and mobile phone-based options, including text messaging and phone apps.

Limitations of this study include a convenience sample and the relatively low response rate with the risk of selection bias. The low response rate means that information was not available from most of the students of interest and it is unknown how well the sample data represent university students in New Zealand [22]. Secondly, the study questions on AoD use were exploratory and have not been previously validated. Thirdly, data were self-reported, and no attempts were made to check the accuracy of these data. Further, the definition of family/whānau or close friend was not specific and may have resulted in some respondents nominating the same person.

This exploratory study provides new data that suggests potentially significant second-hand effects of harmful AoD use on family/whānau and close friends of people who use AoD in New Zealand. University students are vulnerable to the harmful effects of their own AoD use [20,26,33] and of their loved ones and may require support (on and off-campus) to succeed in their studies. The study also provides preliminary data, using novel approaches, that can be used to compare changes in exposure to harmful AoD use among tertiary students. The approaches described in this study (i.e., obtaining information on AoD use from family/whānau members and close friends of people using AoD) should be tested, validated, and used in future research to supplement information collected from traditional in-person surveys with individual AoD users. This would increase the accuracy of data that inform public discourse, and interventions to address the harmful effects of AoD use.

## 5. Conclusions 

Over one-third of respondents reported exposure to family/whānau or close friends in New Zealand who engaged in harmful use of AoD in the previous twelve months (36.2% alcohol; 42.9% another drug), and age and ethnicity were significantly associated with harmful AoD exposures. These results were substantially higher compared with estimates from the general population. The study suggests that data from secondary sources, including family/whanau members and close friends of people using alcohol and other drugs, may be necessary to supplement information from traditional in-person surveys of individual users.

## Figures and Tables

**Table 1:** Demographic characteristics of participants.

	(*n* = 946), %
**Age (years)**	
18–24	751 (79.4)
25–34	152 (16.1)
35 or older	43 (4.5)
**Gender**	
Male	371 (39.2)
Female	555 (58.7)
Other	20 (2.1)
Ethnicity *	
New Zealand European	469 (49.6)
Māori	80 (8.5)
Pasifika	110 (11.6)
Asian	186 (19.7)
Other	228 (24.1)
**Survey type**	
Online	171 (18.1)
Paper	775 (81.9)

* Total may exceed 100% because respondents could choose multiple responses.

**Table 2:** Respondents who had family/whānau member or close friend who consumed alcohol in a harmful way, by questionnaire type.

	Online (*n* = 159) % (95% CI)	Paper (*n* = 714) % (95% CI)	Total (*n* = 873) % (95% CI)	*p*-Value
Yes	40.9 (33.5–48.7)	35.2 (31.7–38.7)	36.2 (33.1–39.4)	0.127
No	56.0 (48.2–63.5)	63.3 (59.7–66.8)	62.0 (58.7–65.1)	
Would rather not say	3.1 (1.4–7.2)	1.5 (0.9–2.7)	1.8 (1.1–3.0)	

**Table 3:** Binary logistic models predicting the likelihood of having family/whānau or close friend who consumed alcohol in a harmful way.

			B	AOR	95% CI	*p*-Value
**Alcohol use vs. No alcohol use**	Age	18–24 years	Ref			
		25–34 years	0.17	1.18	0.77–1.81	0.445
		35 or older	−0.08	0.92	0.47–1.82	0.818
	Gender	Male	Ref			
		Female	−0.14	0.87	0.65–1.18	0.368
		Other	0.17	1.18	0.42–3.37	0.753
	Ethnicity	NZ European	0.50	1.65	0.98–2.77	0.062
		Maori	0.92	2.50	1.50–4.18	<0.001
		Pasifika	0.36	1.44	0.80–2.58	0.227
		Asian	−0.43	0.65	0.37–1.16	0.147
		Other	−0.30	0.74	0.43–1.29	0.286

**Table 4:** Respondents who had family/whānau member or close friend who used drugs in a harmful way.

	(*n* = 798)	%	95% CI
At least one drug including ‘other’	342	42.9	39.5–46.3
Cannabis	261	32.7	29.5–36.0
Synthetic cannabis	39	4.9	3.6–6.6
Ecstasy or MDMA	201	25.2	22.3–28.3
Methamphetamine (Meth, P)	49	6.1	4.7–8.0
Cocaine	44	5.5	4.1–7.3
Heroin	15	1.9	1.1–3.1
Prescription drugs	84	10.5	8.6–12.9
Inhalants (e.g., sniffing glue, lighter fluid, solvents, etc.)	33	4.1	3.0–5.8
Other	43	5.4	4.0–7.2
No, I do not have a family member or close friend who used any of the above	439	55.0	5.6–58.4
Would rather not say	17	2.1	1.3–3.4

**Table 5:** Binary logistic models predicting the likelihood of having family/whānau or close friend who used cannabis, ecstasy/MDMA or prescription drugs in a harmful way.

			B	AOR	95% CI	*p*-Value
**Cannabis use vs. No cannabis use**	Age	18–24 years	Ref			
		25–34 years	0.73	2.08	1.23–3.52	0.006
		35 or older	0.61	1.83	0.80–4.18	0.150
	Gender	Male	Ref			
		Female	0.02	1.02	0.74–1.42	0.889
		Other	0.20	1.22	0.42–3.55	0.711
	Ethnicity	NZ European	1.45	4.26	2.48–7.32	<0.001
		Maori	1.51	4.53	2.55–8.06	<0.001
		Pasifika	0.72	2.06	1.08–3.92	0.028
		Asian	0.17	1.18	0.64–2.19	0.591
		Other	0.82	2.27	1.29–4.00	0.005
**Ecstasy/MDMA use vs. No ecstasy/MDMA use**	Age	18–24 years	Ref			
		25–34 years	1.06	2.88	1.53–5.43	0.001
		35 or older	1.03	2.81	0.96–8.22	0.060
	Gender	Male	Ref			
		Female	0.05	1.05	0.74–1.49	0.803
		Other	0.86	2.37	0.63–8.92	0.201
	Ethnicity	NZ European	1.71	5.50	3.04–9.96	<0.001
		Maori	0.63	1.88	1.06–3.35	0.031
		Pasifika	−0.19	0.83	0.38–1.81	0.640
		Asian	0.11	1.11	0.56–2.21	0.761
		Other	1.00	2.72	1.47–5.03	0.001
**Prescription drug use vs. No prescription drug use**	Age	18–24 years	Ref			
		25–34 years	0.76	2.13	0.89–5.13	0.091
		35 or older	0.31	1.36	0.40–4.61	0.617
	Gender	Male	Ref			
		Female	0.14	1.15	0.72–1.85	0.558
		Other	−0.34	0.71	0.19–2.63	0.609
	Ethnicity	NZ European	0.58	1.79	0.79–4.04	0.161
		Maori	0.36	1.43	0.69–2.97	0.337
		Pasifika	−0.23	0.80	0.29–2.21	0.662
		Asian	−0.44	0.65	0.25–1.70	0.377
		Other	0.09	1.09	0.47–2.54	0.843

## Data Availability

The data are not publicly available due to privacy issues.
